# Estimated COVID-19 Periodicity and Correlation with SARS-CoV-2 Spike Protein S1 Antigenic Diversity, United States

**DOI:** 10.3201/eid3108.250451

**Published:** 2025-08

**Authors:** Erica Billig Rose, Clinton R. Paden, Peter W. Cook, Kevin C. Ma, Amber Winn, Juan Castro, Lakshmi Panagiotakopoulos, Benjamin J. Silk

**Affiliations:** Centers for Disease Control and Prevention, Atlanta, Georgia, USA

**Keywords:** COVID-19, respiratory infections, severe acute respiratory syndrome coronavirus 2, SARS-CoV-2, SARS, coronavirus disease, zoonoses, viruses, respiratory infections, coronavirus, seasons, antigenic variation, spike protein, United States

## Abstract

Emergence of antigenically diverse SARS-CoV-2 variants may be correlated with temporal circulation patterns. We analyzed positive SARS-CoV-2 tests in the United States reported to a national, laboratory-based surveillance network and unique amino acid sequences of the S1 region of the spike protein reported to national genomic surveillance during October 2020–September 2024. We estimated SARS-CoV-2 dominant periodicities using a discrete Fourier transform, described S1 variation using the Simpson diversity index (SDI), and estimated Spearman cross-correlation coefficients between percentage change in SDI and percentage positivity. SARS-CoV-2 activity consistently peaked during July–September and December–February, and dominant periodicities were at weeks 52.2 and 26.1. Percentage positivity and percentage change in SDI were negatively correlated (ρ = −0.30; p<0.001). SARS-CoV-2 peaks occurred in late summer and winter, a pattern likely related to rapid SARS-CoV-2 evolution and cyclical diversity. Monitoring associations between percentage positivity and SDI can help forecast expected surges and optimize prevention and preparedness.

Determining the expected temporal patterns of SARS-CoV-2 circulation has important public health implications, including the timing of vaccine recommendations and health systems preparedness. A single winter peak annually is characteristic of several respiratory viruses, including seasonal influenza, respiratory syncytial virus, and seasonal human coronaviruses (*1*,*2*). Conversely, other respiratory viruses, including parainfluenza and rhinoviruses/enteroviruses, typically peak twice a year (*3*,*4*). Respiratory viruses also have subtype diversity that adds variation into their temporal patterns of circulation (*2*,*4*).

To date, however, few studies that describe SARS-CoV-2 circulation patterns in the United States have been published. One study found the number of reported COVID-19 cases consistently peaked from late fall through spring (*5*). Another study found both an annual winter peak in the number of reported COVID-19 cases and additional periodicity that suggested ≈3 peaks per year (*6*). Such studies confront the challenge of characterizing seasonality relatively soon after emergence of a novel virus and rapid changes in population-level immunity and vaccine introduction that substantially affect virus transmission.

The frequent emergence of new variants, designated phylogenetically, has been characteristic of SARS-CoV-2 evolution, and its antigenic diversity could affect COVID-19 seasonal trends. In the United States, variants of concern detected by genomic surveillance have been temporally associated with increasing COVID-19 incidence to varying degrees. Some variants were temporally associated with mid-year surges observed in late summer 2021 (Delta [B.1.617.2]), late summer 2022 (Omicron BA.5), and late summer 2023 (Omicron EG.5) (*7*–*9*). Other variants were associated with winter surges in 2021–22 (Omicron BA.1), 2022–23 (Omicron XBB.1.5), and 2023–24 (Omicron JN.1), when peaks in respiratory virus activity are expected.

In this study, we sought to determine national and regional SARS-CoV-2 periodicity using the percentage of laboratory detections (percentage positivity) in the United States, which is robust to changes in testing and reporting practices, and thus useful for analyzing temporal patterns of circulation. We also describe SARS-CoV-2 antigenic diversity using the proportion of unique S1 spike genotypes reported each week and the correlation with percentage positivity as a hypothesized driver of COVID-19 seasonality.

## Methods

To determine the percentage of positive SARS-CoV-2 tests reported nationally and by US Department of Health and Human Services (HHS) Region (https://www.hhs.gov/about/agencies/iea/regional-offices), we used data from the National Respiratory and Enteric Virus Surveillance System (NREVSS) during October 2020–September 2024. NREVSS is a voluntary, laboratory-based system to which participating clinical, commercial, and public health laboratories reported the weekly total numbers of aggregate tests performed and SARS-CoV-2 detections. We estimated the proportion of detections that occurred during late summer (July–September) and winter (December–February) and determined the maximum percentage positivity nationally and by HHS Region. We used a discrete Fourier transform (DFT) for spectral decomposition to identify dominant frequencies nationally and regionally; we calculated DFT by using a fast Fourier transform (*10*). We defined dominant periodicities as the reciprocal of the frequencies with the highest magnitude. We then fit those dominant frequencies using linear regression with harmonic functions to model the smoothed, 3-week moving average of the weekly percent positivity data (*6*) (Appendix).

We then determined the weekly predominant SARS-CoV-2 lineages and number of unique amino acid sequences of the S1 region of the spike protein (genotypes) among high-quality sequences submitted publicly for national genomic surveillance during October 2020–September 2024. All sequences included in this analysis are publicly available (https://data.cdc.gov). We estimated variation in the S1 region of the spike protein by using the Simpson diversity index (SDI). SDI is a metric typically used in ecology that incorporates the number and proportion of each species (*11*). Values range from 0 to 1; larger numbers represent more unique sequences with relatively more even distribution (i.e., higher diversity) (Appendix).

We estimated the Spearman cross-correlation coefficient between the percentage change in SDI and smoothed percentage positivity. We selected the lag with the highest mean correlation over 26-week moving windows and estimated the coefficient across both the entire study period and the most recent 2 years, October 2022–September 2024. We computed p values by using the asymptotic *t* approximation (α = 0.05).

## Results

**Figure 1:**
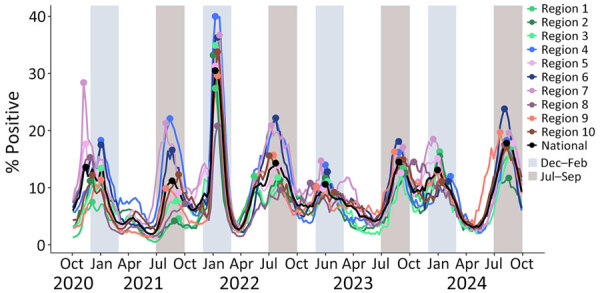
Weekly smoothed (3-week) percentages of positive SARS-CoV-2 tests reported to the National Respiratory and Enteric Surveillance System (NREVSS), nationally and by Health and Human Services (HHS) Region, United States, October 2020–September 2024. The data represent SARS-CoV-2 nucleic acid amplification test results, which include reverse transcription PCR tests from the NREVSS sentinel network of laboratories in the United States, including clinical, public health, and commercial laboratories. These data exclude antigen, antibody, and at-home test results. Blue (December–February) and gray (July–September) vertical bands indicate time periods with increased percentage positivity. Seasonal peaks are indicated by dots at the week when smoothed percent positivity peaked. Regional colors are grouped by geography (e.g., Regions 1, 2, and 3 are shades of green and comprise the Northeast). All HHS Regions had 2 seasonal peaks a year, except HHS Region 8, which only had 1 peak between summer 2023 and winter 2023–2024. HHS Region 1: Connecticut, Maine, Massachusetts, New Hampshire, Rhode Island, and Vermont; HHS Region 2: New Jersey, New York, Puerto Rico, and the Virgin Islands; HHS Region 3: Delaware, District of Columbia, Maryland, Pennsylvania, Virginia, and West Virginia; HHS Region 4: Alabama, Florida, Georgia, Kentucky, Mississippi, North Carolina, South Carolina, and Tennessee; HHS Region 5: Illinois, Indiana, Michigan, Minnesota, Ohio, and Wisconsin; HHS Region 6: Arkansas, Louisiana, New Mexico, Oklahoma, and Texas; HHS Region 7: Iowa, Kansas, Missouri, and Nebraska; HHS Region 8: Colorado, Montana, North Dakota, South Dakota, Utah, and Wyoming; HHS Region 9: Arizona, California, Hawaii, Nevada, American Samoa, Commonwealth of the Northern Mariana Islands, Federated States of Micronesia, Guam, Marshall Islands, and Republic of Palau; HHS Region 10: Alaska, Idaho, Oregon, and Washington. Data from US-affiliated Pacific Islands are not included in NREVSS.

Nationally, SARS-CoV-2 circulated year-round but had peaks in the late summer (July–September) and winter (December–February) months during the entire study period. The only exception occurred during the first winter of the pandemic in 2020–21, when the national peak occurred in late November; during that winter, percentage positivity remained elevated (within 1% of peak positivity) through early January (Figure 1). All HHS regions had peaks 2 times a year, except HHS Region 8, where only 1 peak occurred between summer 2023 and winter 2023–2024. Nearly two thirds (65%; 396,688,638/605,632,990) of detections were reported nationally during the 6 combined months of the late summer and winter periods (i.e., 50% of the year), but the percentages of detections varied by region. HHS Region 4 (the Southeast) had the highest percentage (77%; 60,115,750/78,506,974) reported during late summer and winter, and HHS Region 8 (the Mountain West) had the lowest percentage (54%; 35,198,192/65,255,668). The maximum 3-week smoothed percentage positivity averaged across seasonal peaks varied by region from 13.8% (Region 1) to 22.2% (Region 7). During the most recent 2 years (October 2022–September 2024), the percentage of detections reported nationally during the 6 combined months of late summer and winter was similar to the overall percentage (64%) but showed less regional variation (range 56%–72%).

**Figure 2:**
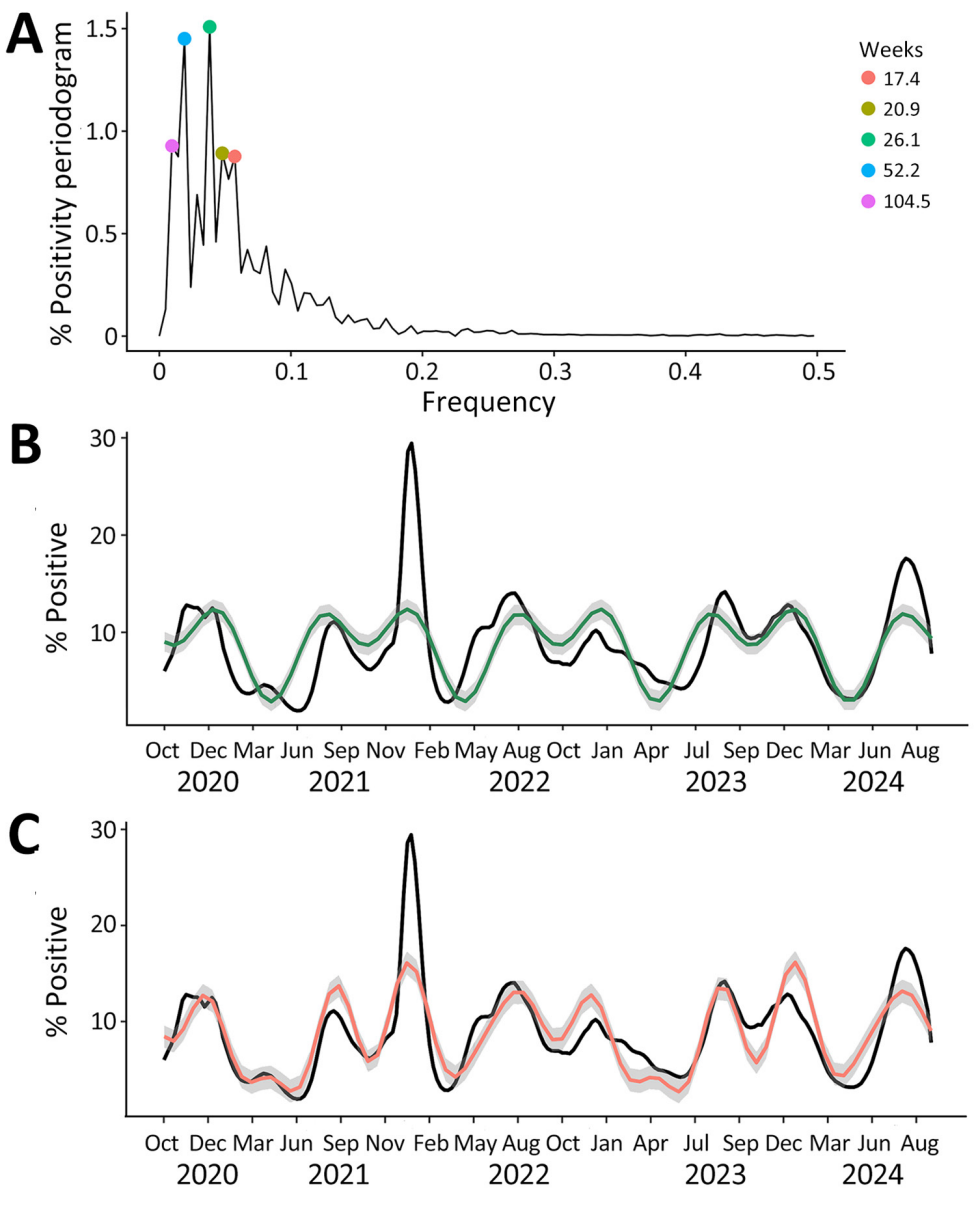
National smoothed (3-week) percentages of positive SARS-CoV-2 tests reported to the National Respiratory and Enteric Surveillance System (NREVSS), United States, October 2020–September 2024. Data represent SARS-CoV-2 nucleic acid amplification test results, which include reverse transcription PCR tests from the NREVSS sentinel network of laboratories in the United States, including clinical, public health, and commercial laboratories. These data exclude antigen, antibody, and at-home test results. A) Periodogram, in which the height of each point indicates strength of the periodicity at the corresponding frequency. Dots indicate 5 dominant periodicities, at frequencies corresponding to surges every 104.5, 52.2, 26.1, 20.9, and 17.4 weeks. Weeks represent time intervals (i.e., weeks do not represent a year of calendar time in the context of this analysis). B) Fitted harmonic function using the 52.2- and 26.1-week periodicities determined by discrete Fourier transform (green line). C) Fitted harmonic function using the 104.5-, 52.2-, 26.1-, 20.9-, and 17.4-week periodicities determined by discrete Fourier transform (pink line).

The spectral decomposition of periodicity revealed 2 nationally dominant periodicities at 52.2 and 26.1 weeks, followed by 3 smaller but notable periodicities at 104.5, 20.9, and 17.4 weeks (Figure 2, panel A). The 2 dominant periodicities fit with observed national trends (R^2^ = 0.43) (Figure 2, panel B), and including the top 5 periodicities improved fit (R^2^ = 0.66), suggesting an overall bimodal seasonality (Figure 2, panel C). Across the 10 HHS Regions, the periodogram varied to include 2–4 of the 7 regionally dominant periodicities; we noted considerable overlap with the nationally dominant periodicities (Appendix Table 1).

**Figure 3:**
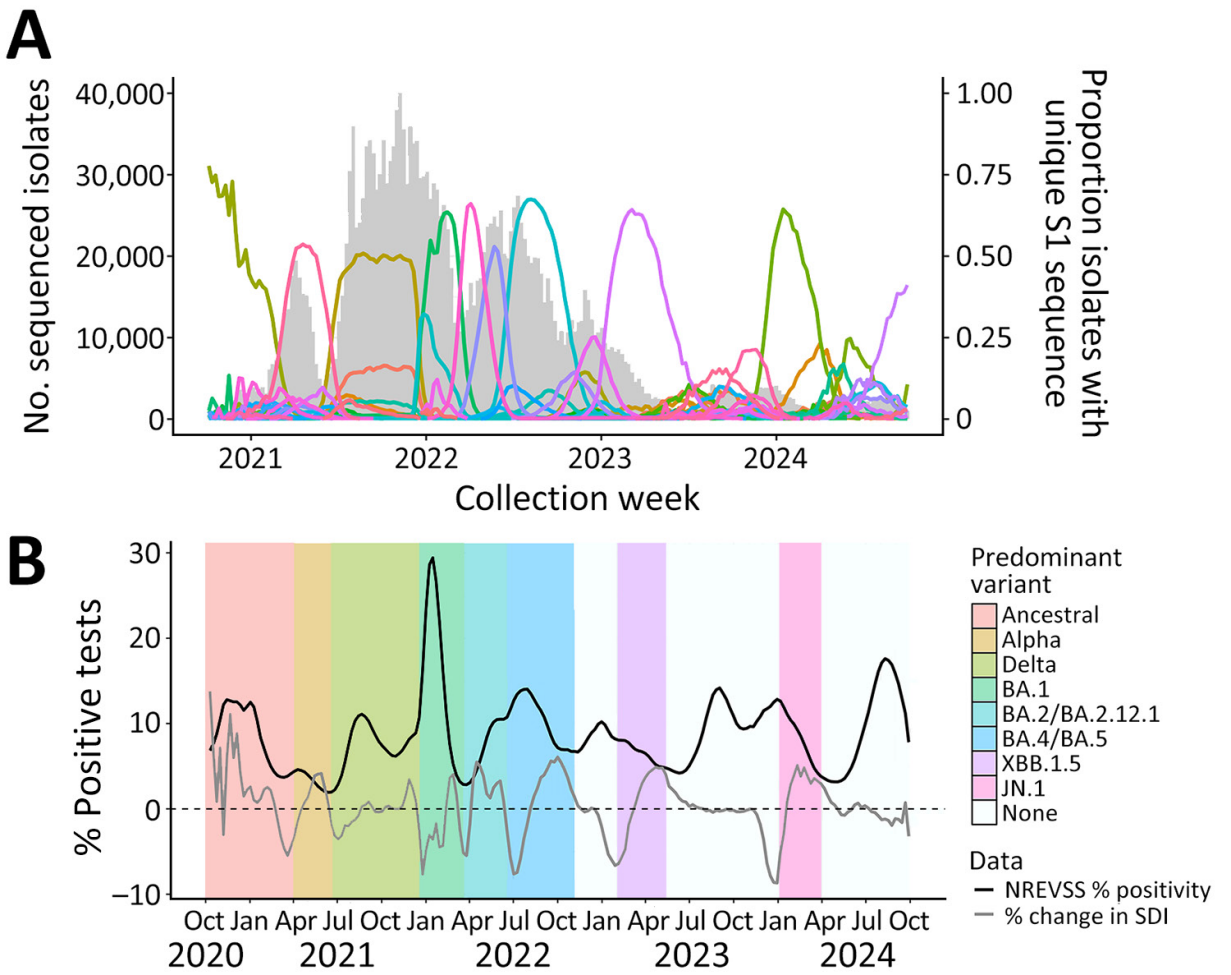
Weekly numbers of sequenced SARS-CoV-2 isolates, proportions with unique S1 spike sequences, and national percentages of positive SARS-CoV-2 tests and percentage changes in SDI, United States, October 2020–September 2024. A) Numbers of sequenced isolates (gray bars) and proportions of isolates with each unique S1 sequence (colored lines). Unique S1 sequences with a maximum proportion of <0.02 during the study period are included in the total number of isolates but not shown as proportion lines to improve visibility of patterns. B) National smoothed (3-week) percentages of positive SARS-CoV-2 tests reported to the NREVSS and percentage changes in SDI of S1 spike proteins. NREVSS, National Respiratory and Enteric Surveillance System; SDI, Simpson diversity index.

We observed periods during which a single S1 sequence predominated among the circulating viruses, often coinciding with seasonal surges and increasing percentage positivity. The volume of sequence data varied from >40,000 high-quality spike sequences per week in 2021 to <1,000 per week in 2023. A single S1 sequence represented >30% of isolates for 46 weeks during 2021 and 43 weeks during 2022; in 2023, we observed only 22 weeks. The predominant S1 sequence consistently had a maximum proportion in the range of 0.5 to 0.7 (Figure 3, panel A).

Periods with a large decrease in SDI, which often represented predominance (>50% prevalence) of a new lineage, typically preceded peaks in percentage positivity, particularly in July 2021 (Delta [B.1.617.2]), December 2021 (Omicron BA.1), July 2022 (BA.4/BA.5), December 2022 (XBB.1.5), and December 2023 (JN.1) (Figure 3, panel B). Percentage positivity and weekly percentage change in SDI had a significant negative correlation maximized at a 2-week lag throughout the entire study period (ρ = −0.30; p<0.001); we observed a stronger correlation during the later part of the study period, starting in October 2022 (ρ = −0.60; p<0.001). The lag suggests that, typically 2 weeks before a peak in percentage positivity, SDI rate of change begins to decrease. However, during the late summer 2023 and 2024 surges, we saw no substantial change in SDI diversity. Instead, several spike S1 sequences with proportions <0.42 circulated; no single predominant variant emerged.

## Discussion

Our analysis revealed biannual COVID-19 peaks in late summer and winter, a pattern that is expected to persist as long as the rapid evolution of SARS-CoV-2 and cyclical S1 diversity continues. The spectral decomposition of periodicity revealed 2 dominant frequencies each year at 52.2 and 26.1 weeks, consistent with a biannual peak approximately every 6 months, and 3 additional notable frequencies at 104.5, 20.9, and 17.4 weeks. Based on the timing of the percentage positivity data, the interval after the winter peak is a longer, 6–9-month period, compared with a 4–5-month interval after the late summer peak. However, our analysis using the DFT approach cannot distinguish whether the periodicity at 20.9 and 17.4 weeks captures an additional, but less apparent, regular seasonality or variation in the timing of the peaks throughout the study period. The periodicity at 104.5 weeks is a harmonic (multiple) of the dominant periodicities that might have been driven by the sharp Omicron BA.1 surge in winter 2021–22, which occurred ≈2 years into the 4-year study period.

A single, dominant S1 sequence genotype was associated with both winter and late summer surges until November 2022. Since winter 2022–23, a dominant genotype was also associated with a surge in virus activity during the winter season, but co-circulating variants with similar spike substitutions have been associated with late summer surges (*12*). Like other betacoronaviruses, SARS-CoV-2 has shown the capacity to undergo both gradual, stepwise evolution and large, periodic changes (*13*,*14*); since 2022, the large shifts in circulating lineages in the United States have occurred during late fall and winter (*12*). The spike protein, and particularly the S1 region, is the major viral protein under selective pressure by population immunity; changes in the spike protein can result in changes in virus fitness or transmission efficiency (*15*). The negative correlation observed between S1 diversity and percentage positivity supports a hypothesis that trends in viral diversity might be a predictor of expected COVID-19 seasonal activity. After surges featuring a predominant spike S1 sequence and associated increasing percentage positivity, the seasonal cycle seems to reset and renewed selective pressure and greater viral evolution subsequently are observed as S1 diversity. Those findings indicate that sustained genomic surveillance sampling during periods of low SARS-CoV-2 activity, when spike diversity is high, is critical for monitoring viral evolution and predicting seasonal increases in SARS-CoV-2 activity, rather than augmenting sequencing when activity is high and antigenic diversity is low. Continued monitoring will be useful for determining whether this cyclical pattern of a predominant spike S1 sequence followed by increased diversity can anticipate future surges in COVID-19 activity.

Transmission of SARS-CoV-2 variants is driven by a combination of intrinsic viral fitness, including cyclical patterns of spike S1 mutations, interacting with a changing landscape of population immunity. After implementation of the national COVID-19 vaccination program in the United States during 2020–2021, natural immunity from infection during the Omicron surge in the winter of 2021–22 further augmented population immunity. SARS-CoV-2 sterilizing immunity after infection typically wanes, although the duration and extent vary after infection with different variants (*16*,*17*). Because vaccine-induced neutralizing antibodies typically wane within 3–6 months, the biannual COVID-19 seasonality highlights the importance of a 2-dose vaccine schedule for older adults and persons with moderate or severe immunocompromise, who are at elevated risk for severe infection (*18*–*20*). In 2025, most of the US population has immune memory from prior infection, vaccination, or both. Studies have shown that a higher number of cumulative SARS-CoV-2 infections and COVID-19 vaccinations leads to higher antibody levels; however, smaller incremental increases in antibody occur with each exposure to the virus or vaccination (*21*,*22*). A study in rhesus macaques determined that high concentrations of neutralizing antibodies alone prevented infection, and those authors calculated an estimated threshold of protection (*23*). In that same study, the authors determined that, near the threshold of immunity after recovery from infection, T cells contributed to viral clearance. In humans, one study estimated that neutralizing antibodies mediated a portion of protection against infection and that the protection is variant specific (*24*). However, those authors hypothesized that nonneutralizing antibodies, likely in the form of cellular immunity, contribute the remaining protection, indicating both humoral and cellular arms of the immune system are important.

The seasonality of many respiratory infections is partly the result of weather and climate; increases in COVID-19 cases have been associated with low temperatures and low humidity (*5*,*25*,*26*). Seasonal patterns of behavior, including school terms, holiday gatherings, and travel, also influence respiratory virus transmission (*27*–*30*). During periods when seasonal surges occur, the risk for exposure increases for persons at higher risk for severe disease. 

The first limitation of this analysis is that the DFT cannot determine why certain frequencies are dominant or whether variations in periodicity throughout the study period occurred; including additional frequencies in the linear regression will continue to improve model fit. Second, different laboratory methods used throughout the study period could have different sensitivities and specificities, causing variation in the reported results of laboratory testing and percent positivity. Third, NREVSS is a passive, voluntary reporting system. Participating laboratories vary from season to season, and SARS-CoV-2 percentage positivity data might not be representative of all geographic areas. Several additional limitations are associated with the changing epidemiologic and clinical features of COVID-19. The proportion of infections causing severe disease has decreased since 2020, which could reduce the number of tests conducted and reported to NREVSS. In addition, the use of screening tests has decreased, and at-home test use peaked in January 2022 (*31*,*32*); those changes can affect how COVID-19 trends are observed and reported. Genomic sampling methods also changed during the study period, and the volume of sequences reported to surveillance decreased. Finally, we described national and regional COVID-19 trends, but local variability in transmission is not represented in our analysis.

In conclusion, SARS-CoV-2 circulation in the United States has been meaningful year-round, but consistent peaks have occurred in late summer and winter since 2020. We observed decreases in antigenic diversity, representing predominance of a new SARS-CoV-2 lineage, before late summer and winter COVID-19 surges during 2021 and 2022. In 2023 and 2024, antigenic diversity increased and remained stable during late-summer surges, indicating sustained co-circulation of multiple lineages. Given the complexity of the interacting factors, additional data and modeling studies are needed to understand the effects of predominant S1 sequences and other drivers of COVID-19 seasonality. Continuing to monitor the associations between percentage positivity and SDI can help to forecast expected surges, describe changing seasonality over time, and optimize prevention and health system preparedness. In particular, the timing of COVID-19 vaccination recommendations could be further optimized to maximize protection according to expected surges in COVID-19 activity. 

Appendix Additional information on estimated COVID-19 periodicity and correlation with SARS-CoV-2 spike S1 antigenic diversity, United States.
