# Supplementary material for: Estimated COVID-19 Periodicity and Correlation with SARS-CoV-2 Spike Protein S1 Antigenic Diversity, United States
Source: Emerg Infect Dis. 2025 Aug;31(8):1573–9. doi: 10.3201/eid3108.250451 (PMC12309744; doi:10.3201/eid3108.250451)
Supplement: Appendix — Additional information on estimated COVID-19 periodicity and correlation with SARS-CoV-2 spike S1 antigenic diversity, United States. [file 25-0451-Techapp-s1.pdf]

Article DOI: https://doi.org/10.3201/eid3108.250451

EID cannot ensure accessibility for supplementary materials supplied by authors. Readers who have difficulty accessing supplementary content should contact the authors for assistance.

Estimated COVID-19 Periodicity and Correlation with SARS-CoV-2 Spike Protein S1 Antigenic Diversity, United States

Appendix

Supplementary Methods

Simpson's Diversity Index (SDI) was defined weekly as the number of isolates with a single S1 sequence, n_i, during week i, among the total number of sequences reported during week i, N_i (*1*):

$$SDI_i = 1 - \frac{\sum n_i\,(n_i - 1)}{N_i(N_i - 1)}$$

To fit the *k* dominant frequencies ω, to the 4-week smoothed percent positivity data, *Y* from weeks 1 to *t*, we used a linear regression as described by Shamsa et al (*2*):

$$Y_i = \sum_{j=1}^{k} \beta_{1j} \cos(2\pi t\omega_j) + \beta_{2j} \sin(2\pi t\omega_j) + e_i$$

References

1. Simpson EH. Measurement of diversity. Nature. 1949;163:688–9. https://doi.org/10.1038/163688a0

2. Shamsa EH, Shamsa A, Zhang K. Seasonality of COVID-19 incidence in the United States. Front Public Health. 2023;11:1298593. PubMed https://doi.org/10.3389/fpubh.2023.1298593

3. Cooley JW, Tukey JW. An algorithm for the machine calculation of complex Fourier series. Math Comput. 1965;19:297–301. https://doi.org/10.1090/S0025-5718-1965-0178586-1

Appendix Table. Dominant periodicities nationally and by HHS Region identified using a discrete Fourier transform of the national 3-week smoothed percentage of positive SARS-CoV-2 tests reported to the National Respiratory and Enteric Surveillance System, October 2020–September 2024*

	Periodicities, wks						
Region†	209	104.5	52.2	34.8	26.1	20.9	17.4
National		X	X		X	X	X
Region 1		X	X		X		X
Region 2		X	X		X		X
Region 3			X		X		X
Region 4	X				X		X
Region 5			X	X	X		X
Region 6			X		X	X	
Region 7			X		X		
Region 8	X		X			X	
Region 9		X			X	X	
Region 10		X			X	X	

*Weeks represent time intervals (i.e., weeks do not represent a year of calendar time in the context of this analysis). The data represent SARS-CoV-2 nucleic acid amplification test results, which include reverse transcription PCR tests from the National Respiratory and Enteric Surveillance System sentinel network of laboratories in the United States, including clinical, public health, and commercial laboratories. These data exclude antigen, antibody, and at-home test results. Calculations based on (*3*).

†HHS Region 1: Connecticut, Maine, Massachusetts, New Hampshire, Rhode Island, and Vermont; HHS Region 2: New Jersey, New York, Puerto Rico, and the Virgin Islands; HHS Region 3: Delaware, District of Columbia, Maryland, Pennsylvania, Virginia, and West Virginia; HHS Region 4: Alabama, Florida, Georgia, Kentucky, Mississippi, North Carolina, South Carolina, and Tennessee; HHS Region 5: Illinois, Indiana, Michigan, Minnesota, Ohio, and Wisconsin; HHS Region 6: Arkansas, Louisiana, New Mexico, Oklahoma, and Texas; HHS Region 7: Iowa, Kansas, Missouri, and Nebraska; HHS Region 8: Colorado, Montana, North Dakota, South Dakota, Utah, and Wyoming; HHS Region 9: Arizona, California, Hawaii, Nevada, American Samoa, Commonwealth of the Northern Mariana Islands, Federated States of Micronesia, Guam, Marshall Islands, and Republic of Palau; HHS Region 10: Alaska, Idaho, Oregon, and Washington. However, data from US-affiliated Pacific Islands are not included in National Respiratory and Enteric Surveillance System.
